# Supplementation of Dietary Quercetin and Vitamin E Promotes the Intestinal Structure and Immune Barrier Integrity in Aged Breeder Hens

**DOI:** 10.3389/fimmu.2022.860889

**Published:** 2022-03-21

**Authors:** Felix Kwame Amevor, Zhifu Cui, Xiaxia Du, Zifan Ning, Xun Deng, Dan Xu, Gang Shu, Youhao Wu, Xueqing Cao, Wei Shuo, Yaofu Tian, Diyan Li, Yan Wang, Yao Zhang, Xiaohui Du, Qing Zhu, Xue Han, Xiaoling Zhao

**Affiliations:** ^1^ Farm Animal Genetic Resources Exploration and Innovation Key Laboratory of Sichuan Province, Sichuan Agricultural University, Chengdu, China; ^2^ Department of Pharmacy, College of Veterinary Medicine, Sichuan Agricultural University, Chengdu, China; ^3^ Guizhou Institute of Animal Husbandry and Veterinary Medicine, Guiyang, China

**Keywords:** chicken, antioxidants, intestinal immunity, inflammation, oxidative stress

## Abstract

In aged animals, the physiological functions of the gastrointestinal tract (GIT) are reduced. Dietary intervention is necessary to re-activate GIT functions. The objective of this study was to investigate the impacts of dietary combination of quercetin (Q) and vitamin E (VE) on the intestinal structure and barrier integrity in aged breeder chickens. A sum of 400 (65-wks-old) Tianfu breeder hens were randomly allotted into four (4) groups with four (4) replicates, and fed with basal diet; basal diet supplemented with 0.4g/kg of Q; basal diet supplemented with 0.2g/kg of VE; and basal diet supplemented with the combination of Q (0.4 g/kg) and VE (0.2 g/kg) for 14 weeks. At the end of the 14^th^ week, serum and gut segments were collected from eight hens per group for analyses. The results showed that Q+VE exerted synergistic effects on intestinal morphology by promoting villi height and crypt depth (P < 0.05), as well as mitigated the intestinal inflammatory damage of the aged hens, but decreased the concentration of serum D-lactate and diamine oxidase; and increased the levels of secretory immunoglobulin A (sIgA) and *Mucin-2* mRNA (P < 0.05). Furthermore, the mRNA expression of intestinal tight junction proteins including *occludin, ZO1*, and *claudin-1* was increased by Q+VE (P < 0.05). Moreover, Q+VE decreased the mRNA expression of the pro-inflammatory genes (*TNF-α, IL-6*, and *IL-1β*), and increased the expression of anti-inflammatory genes (*IL-10* and *IL-4*) (P < 0.05). These results were consistent with the mRNA expression of *Bax* and *Bcl-2*. In addition, Q+VE protected the small intestinal tract from oxidative damage by increasing the levels of superoxide dismutase, total antioxidant capacity, glutathione peroxidase, catalase (P < 0.05), and the mRNA expression of *SOD1* and *GPx-2.* However, Q+VE decreased malondialdehyde levels in the intestine compared to the control (P < 0.05). These results indicated that dietary Q+VE improved intestinal function in aged breeder hens, by protecting the intestinal structure and integrity. Therefore, Q+VE could act as an anti-aging agent to elevate the physiological functions of the small intestine in chickens.

## Introduction

The gastrointestinal tract (GIT) is the largest immunological organ in both animals and humans (1). The gut barrier is made up of several layers which consist of both external anatomical barrier and internal functional immunological barrier systems (2). In addition, tight junction proteins, including occludin, zona occludin-1 and claudins are critical for improving epithelial cell barrier immune functions (3–5). Moreover, the intestinal epithelial barrier interacts with the intestinal components, gut microbiome and immune cells to promote gut immunity (1). However, imbalances in the intestinal microenvironment and other factor such as age could cause disruption in the GIT of animals and humans (3), by reducing the concentration of tight junction proteins, hence, resulting in gut permeability (‘‘leaky gut’’) (1, 1, 6, 7) and several gut disorders, including intestinal inflammatory and autoimmune disorders (4, 8–10). In chickens, oxidative stress is a major factor that disrupts gut structure and integrity (11, 12), because the gut epithelium is prone to oxidative damage (12, 13).

The intestinal antioxidant capacity, gut microbiota and metabolite profile, motility in the intestinal tract regions, GIT transit time and pH, epithelial cell immune function (1, 14), cytokines (15), and intestinal mucus production (16, 17) depend on age and geography, which are influenced by diet (1). Moreover, during aging, there is a high occurrence of bacterial invasion of the intestinal epithelium in animals, thereby disrupting the intestinal barrier function (1, 18), leading to the emergence of gut disorders. Therefore, the intestinal structure and immune barrier integrity are correlated with age and diet (16, 19–21). Moreover, apoptosis of enterocytes and up-regulation of pro-inflammatory cytokines affects the integrity of the intestinal barrier, thereby causing intestinal permeability and hence, reduce the immunoregulatory roles of the intestinal barrier (22, 23). Aschoff et al. (24) reported that enterocytes towards the apex of the villi become increasingly susceptible to apoptosis, and found that *Bax* expression is related to intestinal cell damage (24). hence, alleviating excessive apoptosis and inflammation in the intestine of animals may promote the integrity of the intestinal barrier.

Therefore, a rapid development of dietary supplements to improve gut function and homeostasis in aged animals is imminent (25, 26). Vitamin E (VE) is a powerful dietary supplement characterized with a strong antioxidant property that attenuates the production and accumulation of reactive oxygen species (ROS) to prevent tissue damage caused by oxidative stress in chickens (27, 28). It is involved in muscular activity, tissue integrity and immune response by hindering the production of reactive oxygen species (29–33). Studies have shown that VE supplementation improves intestinal structure and mitigates intestinal inflammation in chickens (26). Moreover, VE has a synergistic effect with other dietary supplements such as flavonoids (27, 28, 34), selenium (35), and alpha lipoic acid (26, 36) to enhance antioxidant activity in animals. Vitamin E (alpha- and gamma-tocopherol) supplementation was reported to mitigate colitis, as well as protect the intestinal barrier function in mice by inhibiting colitis-induced loss of the tight junction protein occludin, and mitigates TNF-α/IFN-γ-induced impairment of trans-epithelial electrical resistance in human intestinal epithelial Caco-2 cell monolayer (37). In addition, supplementing vitamin E reduces phoxim (organophosphate pesticides) toxicity in rat intestinal tissues by alleviating phoxim-induced toxic effects on the intestinal oxidative stress, barrier function, and morphological changes (38). Furthermore, a study by Cadir et al. (39) showed that the supplementation of omeprazole and/or vitamin E exerts protective effects on the biochemical and histopathological intestinal damage induced by hypoxia/reoxygenation in newborn rats (39).

Dietary flavonoids such as catechin, resveratrol, rutin, and quercetin (Q) are polyphenolic compounds which are universally present in plants, and play major roles in chicken performance (40–42). Quercetin is a polyphenol belonging to the class of flavonoids obtained from fruits, vegetables, and beverages (27, 43). It is a potent antioxidant that enhances intestinal barrier function. Reports have indicated that quercetin promotes the concentration of tight junction proteins including claudin-1, claudin-4, occludin, and Zona occludin-1 (44, 45), thereby promoting barrier function and reducing inflammation. Furthermore, quercetin enhances barrier integrity in Caco-2 cells by inducing remodeling of epithelial tight junctions, as well as protects gastric mucosa against ulcerogenic agents (44). Yan et al. (46) reported that quercetin exhibits protective effect on indomethacininduced gastric mucosal injury in rats, by inducing mucus secretion (46). Quercetin also attenuates the effects of *C. rodentium*-induced colitis, inhibit the production of pro-inflammatory cytokines, as well as upregulates the expression of anti-inflammatory cytokines in the colon (47). In a high-fat-diet-fed mice model, quercetin exerts protective effect against immune/inflammatory responses and oxidative stress, and decreased intestinal lipid levels by attenuating atherosclerotic lesions (48). Moreover, in broiler chickens, quercetin supplementation alleviates oxidative stress and mitochondria damage induced by lipopolysaccharide (LPS)-induce *via MAPK/Nrf2* signaling in the intestine, thereby increases the villus height and crypt depth in the duodenum, jejunum, and ileum (49). In addition, quercetin improves the intestinal health by decreasing serum endotoxin levels, reduces the intestinal ROS production, and increases the jejunal villi height and upregulated the mRNA expression of *occludin* and *zonula occudens-1* in the jejunum of finishing pigs (50), as well as attenuates colon damage by upregulating the expression of *Muc2* and *ZO-1* in C57BL/6J mice (51).

Reports indicated that quercetin supplementation increased the intestinal villi length, as well as enhanced the mucosal thickness of the intestine (52), and restored barrier function in antibiotic-treated mice through decreasing the expression of serum biomarkers such as D-lactic acid and serum diamine oxidase levels (53).

Dong et al. (54) reported that quercetin supplementation upregulated the mRNA expression of tight junction proteins (such as *tight junction protein 1*) and *Mucin-2* in chickens (54). These studies have provided the basic evidence for the role of quercetin as an important supplement that can promote intestinal barriers function in animals. Becker et al. (34) reported that quercetin has a strong synergistic effect with other dietary supplements such as rutin and *α*-tocopherol without any detrimental effects (34). Moreover, quercetin has also been found to have interactive effects with dietary supplements including catechin (55), citrulline (25), and resveratrol (40) to promote physiological functions in animals.

However, to the best of our knowledge, no studies have reported the interactive effects of quercetin and vitamin E on gut function in aged animals. Hence, the aim of this current study was to determine the impacts of dietary quercetin and vitamin E, supplemented independently and in combination on the intestinal structure and immune barrier integrity of aged breeder hens.

## Materials and Methods

### Birds, Management, and Experimental Design

A total of 400 Tianfu Breeder Hens (65 weeks old) obtained from the Chicken Breeding Unit, Sichuan Agricultural University (the characteristics of this chicken breed were described in our previous study (27) were randomly allotted into 4 treatments containing 100 birds each, with 4 replicates of 25 chickens each. The birds were housed in individual wire cages (width: 48.8 cm, depth: 38.1 cm, height: 38.1 cm) and the lighting system was controlled (16 h light per day) and optimal ventilation was maintained throughout the experiment. Quercetin (95%, High-Performance Liquid Chromatography (HPLC) and Vitamin E were supplied by Shaanxi Huike Plant Development Co., Ltd. (Xian, Shaanxi, China). We determined the purity (95%) of the quercetin using HPLC. The chickens were fed a basal diet (control group); basal diet supplemented with 0.4 g/kg quercetin powder (quercetin group); basal diet supplemented with 0.2 g/kg vitamin E (vitamin E group); and basal diet supplemented with the combination of 0.4 g/kg quercetin and 0.2 g/kg vitamin E (Q + VE group). The recommended levels of vitamin E and quercetin inclusion were chosen based on previous studies (56) and (43, 57), respectively. Throughout the experimental period, the hens were given 120 g feed/day/hen, under a photoperiod of 16L:8D, and water was provided *ad libitum*. The ingredient composition in percentages and the calculated nutritional values of the basal diet fed the aged hens have been reported (27). The feed intake and body weight of the birds were measured weekly throughout the experimental period. The average body weight and average daily feed intake were calculated.

### Sample Collection and Procedure

The experiment lasted for 14 weeks, and at the end of the 14^th^ week, we randomly selected 2 birds per replicate (8 birds per treatment, totaling 32) whose blood samples (5 mL) were collected *via* the wing vein. The blood samples were centrifuged at 3,000 rpm for 10 min at 4°C to obtain the serum, and then stored at -80^0^C for further analyses. Subsequently, the selected chickens were euthanized, their viscera were excised, the intestine was discretely separated and the adherent materials were precisely removed. The intestinal segmental samples (duodenum, jejunum, and ileum: thus, the duodenum from the ventriculus to the pancreatobiliary ducts; jejunum from pancreo-biliary ducts to yolk stalk; and ileum from yolk stalk to ileocecal junction) were collected, and after squeezing out the contents, the remaining small intestine was cut and the mucosa was gently scraped using a clean glass slide, immediately frozen in liquid nitrogen, and stored at -80°C for subsequent RNA extraction and qRT-PCR analysis. Moreover, parts of the intestinal mucosa (duodenal, jejunal, ileal mucosa) were stored at -20°C for subsequent biochemical analysis. The middle portions of the three sections of the small intestine (duodenum, jejunum, and ileum) were collected, washed in PBS, and fixed in 4% paraformaldehyde for morphological analysis.

### Biochemical Analysis

The intestinal mucosa obtained from the, duodenal, jejunal, and ileal tissues were homogenized in pre-cold 0.9% saline and centrifuged at 3,000 rpm for approximately 10 min at 4°C to obtain the supernatant. Later, the supernatant was stored at -20°C for biochemical analysis. In addition, the protein concentration in the supernatant was determined using a Total Protein Assay kit according to the manufacturer’s instructions (Nanjing Jiancheng Bioengineering Institute, Nanjing, China). Below are the parameters measured in the serum and intestinal mucosa (duodenal, jejunal, and ileal) tissue supernatants.

### Serum DAO Activity and D-Lac Concentration (Intestinal Barrier Biomarkers)

The activity and concentration of the serum diamine oxidase (DAO) and D-lactate were determined using the enzyme-linked immunosorbent assay (ELISA) kits following the manufacturer’s instructions (Baolai Biotechnology Co., Ltd., Yancheng, China).

### Antioxidant Status

The activities of superoxide dismutase (SOD), total antioxidant capacity (TAOC), glutathione peroxidase (GPx), catalase (CAT), and malondialdehyde (MDA) in the intestinal mucosa supernatants and plasma were determined using commercial biochemistry kits according the instructions provided by the manufacturer (Nanjing Jiancheng Bioengineering Institute, Nanjing, China). The parameters (TAOC, CAT, GPx, and SOD levels) determined in the tissue (intestinal) homogenates were expressed as units per milligram of protein, whereas the levels of the MDA was expressed as nanomoles per milligram of protein.

### Biochemical Analysis of the Concentration of Cytokines and sIgA Levels in the Plasma and Small Intestinal Mucosa

The expression levels of cytokines such as IL-1β, IL-6, IL-4, and IL-10, were measured in the supernatants of the intestinal mucosa (ileum, duodenum, and jejunum) and plasma using commercial biochemistry kits following the protocols provided by the manufacturer (Nanjing Jiancheng Bioengineering Institute, Nanjing, China). Moreover, the concentration of secretory IgA (sIgA) was determined in the intestinal mucosa (duodenum, jejunum, and ileum) using ELISA kits according to the guide provided by the manufacturer (Baolai Biotechnology Co., Ltd., Yancheng, China).

### Intestinal Histomorphology

For morphological analysis, the duodenal, jejunal, and ileal tissue samples were stored in 4% paraformaldehyde for 48 h. Thereafter, we prepared the samples using paraffin embedding techniques. The sections (5µm) were stained using hematoxylin and eosin (HE) for morphological and structural observation. The dyed slices of the duodenum, jejunum, and ileum were used to examine the pathological status of the intestinal tissues under a light microscope (DP80Digital, Olympus, Tokyo, Japan) and images were captured. Furthermore, the morphological characteristics such as villus height (from the tip of the villus to the crypt opening/junction) and crypt depth (from the opening of the invagination to the base above the lamina muscularis mucosae, i.e., distance of the invagination between 2 adjacent villi) were measured using ImagePro Plus 6.0 software (Media Cybernetics). At least ten (10) views were selected from each intestinal sample for measurement (thus, 10 separate well-oriented villi and crypts were measured per slide), and the average of each index was recorded. Furthermore, we grade (score) the degree of duodenum, jejunum, and ileum tissue damage in accordance with the degree of inflammation described in previous studies (58, 59). Thus, the severity of the histological inflammation were scored as follows: 0 = none; 1 = mild; 2 = moderate; and 3 = severe. In addition, the inflammatory cell infiltration were scored as: 0 = normal; 1 = mucosal; 2 = submucosal; and 3 = osmotic transmural expansion, whereas, the scores for the epithelial lesions were; 0 = complete; 1 = crypt structure deformation; 2 = erosion; and 3 = ulcers. Moreover, the grades for the extent of lesions were; 0 = none; 1 and 2 = multifocal; and 3 = spread, while, the scores for edema were 0 = none; 1 = mild mucosal; 2 = submucosal; and 3 = mucosal). In all, these scores were computed to determine the damage scores for the various intestinal tissues.

### RNA Extraction and Real-Time Quantitative PCR

Total RNA was extracted from the intestinal (duodenum, jejunum, and ileum) mucosal scrapings following previously described procedures (27, 28, 60), using TRIzol reagent (Takara, Dalian, China), according to the manufacturer’s instructions. Then, the concentration and purity of the extracted RNA were determined using Nanodrop 2000C (Thermo Fisher Scientific, Waltham, MA, USA) with an absorbance ratio of A260/280. Thereafter, the PrimeScript RT Reagent Kit (Takara, Dalian, China) was used to synthesize the singlestrand cDNA following the protocols provided by the manufacturer. Thereafter, we used the single-strand cDNA for the qRT-PCR analysis using the CFX96 Real-Time System (Bio-Rad, Hercules, CA, USA) under favorable conditions such as: 95°C for 3 min, 40 cycles of 95°C for 10 s and annealing temperature (**Table 1**) for 20 s, which was followed by a final extension at 72°C for 20 s, with a melt curve analysis performed at 65~95°C. The amplification efficiencies of the target genes ranged from 95% to 105%. We have performed each qRT-PCR reaction with the volumes of 15 µL containing 6.25 µL TB Green TM Premix (Takara), 0.3 µL forward and reverse primers, 1.5 µL cDNA, and 6.65 µL DNase/RNase-Free Deionized Water (Tiangen, Beijing, China). The samples were run in triplicate, and the expression level of *β-actin* was used to normalize the cycle threshold (Ct) values. The relative abundance of each transcript was normalized to that of *β-actin*. Gene expression was calculated using the 2*^−^* ^∆∆Ct^ method (61).

**Table 1:** Primers used for quantitative real-time polymerase chain reaction (qRT-PCR).

Genes	Sequence (5’-3’)	Product Length (bp)	Annealing Temperature (℃)	Accession Number
***Gut structure and barrier related genes***				
*** Claudin 1***	F: GACCAGGTGAAGAAGATGCGGATG R: CGAGCCACTCTGTTGCCATACC	107	59.17	NM_001013611.2
*** Zona occludens 1***	F: CTTCAGGTGTTTCTCTTCCTCCTC R: CTGTGGTTTCATGGCTGGATC	131	59.82	XM_015278981.2
*** Occludens***	F: TCATCGCCTCCATCGTCTAC R: TCTTACTGCGCGTCTTCTGG	240	57.79	XM_025144248.1
*** Mucin 2***	F: CCCTCACCCAGCCCGACTTC R: GCCGTTGGTGGAGGTGTTACAG	179	58	JX284122.1
***Antioxidant related genes***				
***SOD1***	F: GGCAAGCAGCACGGTGGAC R: CTTCTGCCACTCCTCCCTTTGC	129	59	NM_205064.1
*** GPx2***	F: ACGGCACCAACGAGGAGATCC R: CTTCCCGTTCACCTGGCACTTC	175	60.67	NM_001277854.2
***Pro- and anti-inflammatory genes***				
* TNF* ***-*** *α*	F: TGTGCTGTGTGCAACGACTA R: CAGGCCTGGCAACTCTTTCT	167	57	NM_205183.2
*** IL-6***	F: CTGCAGGACGAGATGTGCAA R: AGGTCTGAAAGGCGAACAGG	175	60.67	NM_204628.1
* IL-1β*	F: TGCCTGCAGAAGAAGCCTCG R: GACGGGCTCAAAAACCTCCT	204	60.25	NM_204524.1
*** IL-10***	F: GGAGAGAGCGGAGGTTTCG R: TCCCGTTCTCATCCATCTGC	119	59.86	XM_025143715.1
*** IL-4***	F: ACATCCAGGGAGAGGTTTCCT R: GTGGGACATGGTGCCTTGAG	208	60.20	NM_001007079.1
***Apoptosis genes***				
*** Bcl-2***	F: ATCGTCGCCTTCTTCGAGTT R: ATCCCATCCTCCGTTGTCCT	150	59	Z11961.1
*** Bax***	F: GTGATGGCATGGGACATAGCTC R: TGGCGTAGACCTTGCGGATAA	90	58	XM_422067.4
***Housekeeping gene***				
*** β-actin***	F: ATCCGGACCCTCCATTGTC R: AGCCATGCCAATCTCGTCTT	120	60	NM_205518.1

### Statistical Analysis

All data were analyzed by one-way analysis of variance (ANOVA) using GraphPad Prism version 6.01 statistical package for Windows (GraphPad Software Inc., San Diego, CA) and SPSS 20 Statistical Analysis Software (SPSS Inc., Chicago, IL, USA). Therefore, all the experimental data are indicated as the mean ± standard deviation (SD), and Tukey’s test was used to determine the differences among the treatment groups. Calculated Δ Ct (corrected sample) = mean value of target gene - mean value of internal reference gene, ΔΔ Ct = Δ Ct-mean value of control group. Values were significantly different at P < 0.05.

## Results

### Effects of Dietary Quercetin, Vitamin E, and Q + VE on Feed Intake and Body Weight

**Figure 1:**
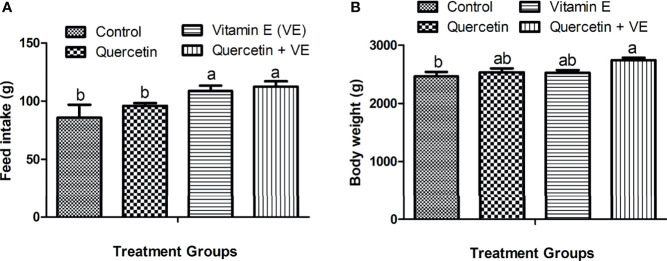
Effects of quercetin (Q), vitamin E (VE), and Q + VE on feed intake and body weight of aged laying hens fed 14 weeks. Bars without the same letter differed significantly (P < 0.05). **(A)** Feed intake; **(B)** body weight.

In this study, we observed the average daily feed intake and body weight per hen after 14 weeks of the experimental period. The feed intake recorded for the aged laying hens was significantly improved by the combination of Q + VE which is similar to that recorded in the VE group but differed from the Q and control groups (**Figure 1A**, P < 0.05). In addition, the body weight of the Q + VE group was significantly higher than those of the control group (**Figure 1B**, P < 0.05).

### Effects of Quercetin, Vitamin E, and Q + VE on the Activities of Serum Diamine Oxidase and D-lactate (Intestinal Biomarkers)

**Figure 2:**
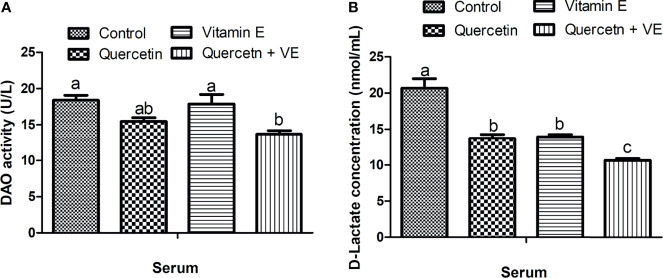
Effects of quercetin (Q), vitamin E (VE), and Q + VE on the activities of serum diamine oxidase (DAO) and D-lactate (D-LA) (intestinal biomarkers) after 14 weeks experimental feeding. Bars without the same letter differed significantly (P < 0.05). **(A)** serum DAO; **(B)** D-LA.

As shown in **Figure 2**, the concentration of serum diamine oxidase (DAO) in the Q + VE group was lower than that in the control and VE groups (**Figure 2A**, P < 0.05). In addition, the serum D-lactate (D-LA) level was significantly lower in the Q + VE group than in the other groups (**Figure 2B**, P<0.05). The Q and VE groups were significantly different from those of the control group (**Figure 2B**, P < 0.05). These results indicated that the combination of Q + VE had a synergistic effect on the intestinal barrier biomarker indices of aged breeder hens.

### Effects of Quercetin, Vitamin E, and Q + VE on Antioxidant Indices in the Serum and Intestinal Mucosa

**Table 2** summarizes the antioxidant indices in the intestinal segments (duodenum, jejunum, and ileum) and serum. The concentration of SOD in the serum was significantly higher in the Q + VE group than in the other groups (P < 0.05). In addition, there was a significant difference in the levels of SOD in the duodenum between the Q + VE and control groups. Meanwhile, the SOD level in the jejunum of the Q + VE group was significantly higher than those in the control and VE groups (P < 0.05). Interestingly, the ileal SOD level in the Q + VE group was significantly higher than that in the other groups (P < 0.05). Furthermore, the TAOC level in the serum of the Q + VE group was also higher than those in the other groups (**Table 2**; P < 0.05). The TAOC concentration in the duodenum was significantly different between the Q + VE and control groups (P < 0.05). In addition, serum GPx was significantly improved in the Q and Q + VE groups compared to the control group (p < 0.05). Again, the activity of GPx in the duodenum was increased by Q + VE compared with that in the control group (P < 0.05). Moreover, the level of GPx in the jejunum was improved by both the Q and Q + VE groups compared to the control and VE groups (P < 0.05). However, the Q + VE group had a higher concentration of GPx in the ileum than the other groups (P < 0.05). The CAT level in the serum was also increased by dietary Q + VE compared to the control and Q groups (P < 0.05). In the duodenum, CAT was significantly increased by the Q, VE, and Q + VE dietary treatments compared to the control group (P < 0.05). Moreover, CAT levels in the jejunum were significantly higher in the Q and Q + VE groups than in the control group (P < 0.05). Moreover, the Q + VE group was significantly higher than that of the control group (P < 0.05). Interestingly, the MDA levels in the serum, duodenum, jejunum, and ileum were significantly higher in the control group than in the other groups (P < 0.05).

**Table 2:** Effects of Q, VE, and Q + VE on antioxidant enzymes and MDA levels in the intestinal segments, and serum of aged breeder hens.

Parameters^1^	Tissues	Treatments				
		Control	Q	VE	Q+VE	P-value
SOD	Serum	15.86^b^ ± 0.48	17.51^b^ ± 0.80	15.90^b^ ± 0.73	28.43^a^ ± 3.87	0.001
	Jejunum	15.55^b^ ± 0.85	20.62^ab^ ± 2.97	17.93^b^ ± 0.91	27.04^a^ ± 1.32	0.001
	Duodenum	22.01^b^ ± 1.55	27.23^ab^ ± 2.57	25.39^ab^ ± 2.29	30.95^a^ ± 1.78	0.030
	Ileum	16.85^b^ ± 0.77	19.61^b^ ± 3.14	16.64^b^ ± 0.61	28.15^a^ ± 3.08	0.002
TAOC	Serum	16.01^b^ ± 0.48	17.79^b^ ± 0.61	17.73^b^ ± 0.97	20.76^a^ ± 1.34	0.001
	Jejunum	20.46^b^ ± 1.13	26.29^ab^ ± 1.88	25.46^ab^ ± 2.36	29.11^a^ ± 1.51	0.010
	Duodenum	10.90^b^ ± 0.34	16.02^ab^ ± 0.86	15.74^ab^ ± 0.75	17.96^a^ ± 2.50	0.005
	Ileum	15.18^b^ ± 0.70	18.18a^b^ ± 1.35	17.57^ab^ ± 0.94	21.15^a^ ± 0.70	0.001
GPx	Serum	15.75^c^ ± 1.05	19.79^ab^ ± 0.85	17.02^bc^ ± 0.60	22.51^a^ ± 1.18	0.001
	Jejunum	40.90^b^ ± 1.44	56.12^a^ ± 1.93	42.41^b^ ± 2.52	58.99^a^ ± 2.33	0.001
	Duodenum	19.52^b^ ± 0.69	23.01^ab^ ± 1.64	21.19^ab^ ± 1.45	25.84^a^ ± 1.86	0.025
	Ileum	34.56^b^ ± 1.84	38.04^b^ ± 1.53	36.54^b^ ± 1.63	46.04^a^ ± 1.85	0.001
CAT	Serum	14.68^b^ ± 0.84	15.34^b^ ± 0.77	16.65^ab^ ± 0.68	18.81^a^ ± 0.77	0.002
	Jejunum	45.53^c^ ± 1.87	55.40^ab^ ± 2.72	49.63^bc^ ± 2.18	63.73^a^ ± 2.68	0.001
	Duodenum	34.25^c^ ± 2.69	43.59^b^ ± 1.19	48.74^ab^ ± 1.46	52.28^a^ ± 1.97	0.001
	Ileum	18.31^b^ ± 0.62	21.01^ab^ ± 1.19	21.18^ab^ ± 1.59	26.33^a^ ± 2.09	0.003
MDA	Serum	23.60^a^ ± 1.69	15.27^b^ ± 1.02	14.81^b^ ± 0.65	14.26^b^ ± 0.87	0.001
	Jejunum	1.86^a^ ± 0.11	1.09^b^ ± 0.12	1.03^b^ ± 0.11	1.00^b^ ± 0.10	0.001
	Duodenum	1.61^a^ ± 0.07	0.99^b^ ± 0.14	1.09^b^ ± 0.13	0.75^b^ ± 0.10	0.001
	Ileum	1.55^a^ ± 0.14	0.91^b^ ± 0.13	0.93^b^ ± 0.12	0.70^b^ ± 0.08	0.001

^abc^Means within 4 treatments (control, quercetin, vitamin E, and Q + VE) lacking a common superscript differed significantly (P < 0.05).

CAT, catalase; Ctrl, control group; GPx, glutathione peroxidase; MDA, malondialdehyde; SOD, superoxide dismutase; TAOC, total antioxidant capacity; prot, protein. ^1^ SOD, TAOC, GPx, and CAT: U/mg of protein (nmol/mg prot.) in intestinal tissues and U/mL in serum. MDA: nmol/mg of protein in intestinal tissues; MDA: nmol/mL in serum.

### Effects of Quercetin, Vitamin E, and Q + VE on the Biochemical Levels of Intestinal Mucosa and Plasma Cytokines (IL-6, IL-1β, IL-10, and IL-4)

**Table 3** summarizes the expression levels of pro-inflammatory (IL-6 and IL-1β) and anti-inflammatory cytokines (IL-10 and IL-4) in the intestinal mucosa and serum. The levels of pro-inflammatory cytokines (IL-6 and IL-1β) were significantly reduced in the Q + VE treatment group as compared with the control group (P < 0.05). However, in some instances, the individual Q and VE groups reduced the concentrations of these pro-inflammatory cytokines (IL-6 and IL-1β) in the intestinal mucosa and serum; for example; IL-1β was reduced in the three gut segments by the individual Q and VE. However, in some cases the individual Q and VE groups were similar to both the control and Q + VE groups (P > 0.05) across all the intestinal tissues (duodenum, jejunum, and ileum mucosa) and plasma tested. Moreover, the levels of the anti-inflammatory cytokines (IL-10 and IL-4) were highly elevated in the Q, VE, and Q + VE groups (with the highest level in the Q + VE group) compared with the control group (P < 0.05). This shows that the combination of Q + VE exerts synergistic effects in attenuating intestinal and systemic inflammation in aged breeder chickens.

**Table 3:** Effects of supplemental Q, VE, and Q + VE on pro- and anti-inflammatory cytokines in the intestinal mucosa and plasma of the aged breeder hens.

Cytokines^1^	Tissues	Treatments				
		Control	Q	VE	Q + VE	P-value
** IL-1**β	Serum	36.59^a^ ± 2.05	25.96^b^ ± 1.32	26.05^b^ ± 1.60	20.44^b^ ± 1.94	0.001
	Jejunum	44.22^a^ ± 1.64	33.46^b^ ± 1.65	37.30^b^ ± 2.04	22.36^b^ ± 1.78	0.011
	Duodenum	46.09^a^ ± 2.36	42.83^b^ ± 1.78	36.44^b^ ± 1.67	30.42^b^ ± 1.70	0.001
	Ileum	46.97^a^ ± 2.90	32.21^b^ ± 1.69	32.44^b^ ± 1.93	30.42^b^ ± 2.65	0.003
** IL-6**	Serum	44.73^a^ ± 2.83	38.33^b^ ± 1.94	37.17^b^ ± 1.28	31.17^b^ ± 1.69	0.001
	Jejunum	42.22^a^ ± 1.83	31.46^b^ ± 2.32	29.67^b^ ± 2.40	26.66^b^ ± 2.57	0.001
	Duodenum	50.35^a^ ± 1.87	40.41^ab^ ± 2.01	42.44^ab^ ± 1.75	36.66^b^ ± 1.28	0.001
	Ileum	43.48^a^ ± 2.18	31.81^b^ ± 2.30	32.72^b^ ± 1.85	29.79^b^ ± 1.95	0.001
** IL-4**	Serum	27.86^c^ ± 1.32	38.35^b^ ± 1.34	39.12^b^ ± 2.01	48.40^a^ ± 1.80	0.001
	Jejunum	30.31^b^ ± 2.07	40.82^a^ ± 1.89	43.92^a^ ± 1.96	48.40^a^ ± 1.96	0.006
	Duodenum	37.19^b^ ± 1.88	46.31^ab^ ± 2.09	42.50^ab^ ± 2.66	53.47^a^ ± 2.34	0.001
	Ileum	35.94^b^ ± 2.94	47.83^ab^ ± 2.24	46.00^ab^ ± 3.98	49.65^a^ ± 2.44	0.009
** IL-10**	Serum	26.56^c^ ± 1.90	35.19^ab^ ± 1.27	39.41^bc^ ± 1.37	40.41^a^ ± 1.22	0.007
	Jejunum	30.93^b^ ± 1.83	43.94^ab^ ± 1.96	41.99^ab^ ± 1.70	48.00^a^ ± 1.63	0.001
	Duodenum	35.31^c^ ± 1.64	43.36^b^ ± 1.75	48.49^ab^ ± 1.88	50.25^a^ ± 1.57	0.001
	Ileum	23.42^c^ ± 2.09	42.07^b^ ± 1.83	43.74^b^ ± 1.88	56.06^a^ ± 2.16	0.001

^abc^Means within 4 treatments (control, quercetin, vitamin E, and Q + VE) lacking a common superscript differed significantly (P < 0.05).

IL-1**β**, Interleukin-1**β**; IL-6, Interleukin-6; IL-4, Interleukin-4; IL-10, Interleukin-10, prot: protein. ^1^IL-1**β**, IL-6, IL-4, and IL-10: U/mg of protein in intestinal tissues and U/mL in serum.

### Effects of Dietary Quercetin, Vitamin E, and Q + VE on the sIgA Levels (Immune Biomarker) in the Mucosa of Duodenum, Jejunum, and Ileum

The activities of sIgA in the small intestinal mucosa are summarized in **Table 4**. The concentration of sIgA in the duodenum, jejunum, and ileum of the aged chickens was significantly increased in the Q, VE, and Q + VE groups compared to the control group (P < 0.05). However, in all small intestinal segments, sIgA levels were similar in the Q, VE, and Q + VE groups (P > 0.05).

**Table 4:** Effects of supplemental Q, VE, and Q + VE on the immune biomarker (sIgA) levels in the intestinal mucosa of the aged breeder hens.

Parameter^1^	Treatments^1^					
	Tissue	Control	Q	VE	Q+VE	P-value
** sIgA**	Jejunum	9.30^b^ ± 0.56	17.02^a^ ± 1.90	20.41^a^ ± 2.81	21.06^a^ ± 1.99	0.001
	Duodenum	9.23^b^ ± 0.63	14.56^a^ ± 1.51	15.54^a^ ± 1.23	16.56^a^ ± 1.13	0.001
	Ileum	14.55^b^ ± 1.04	21.87^a^ ± 2.44	24.00^a^ ± 2.81	23.73^a^ ± 1.86	0.026

^ab^Means within 4 treatments (control, quercetin, vitamin E, and Q + VE) lacking a common superscript differed significantly (P < 0.05).

^1^sIgA, secretory immunoglobulin A; prot, protein.

### Effects of Quercetin, Vitamin E, and Q + VE on the mRNA Expression of the Tight Junction Proteins and Barrier Function Biomarker Gene in the Chicken Intestinal Mucosa

**Figure 3:**
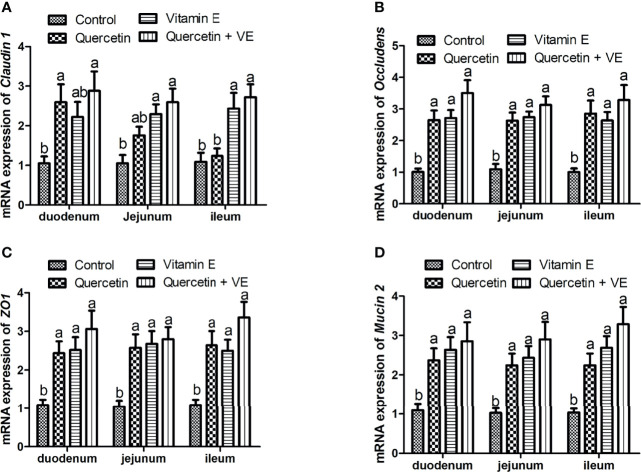
The impacts of quercetin (Q), vitamin E (VE), and Q + VE on the mRNA expression of the tight junction proteins and barrier function biomarker genes in the small intestinal mucosa of the aged breeder hens. mRNA expression of: **(A)** occludin; **(B)** claudin 1; **(C)** ZO1; and **(D)** Mucin 2 in the small intestinal mucosa. Bars without the same letter differed significantly (P < 0.05).

As shown in **Figure 3**, the mRNA expression of the tight junction proteins [*occludin, zona occludens 1* (*ZO1*), and *Claudin 1*] and barrier function (*Mucin 2*) related genes were significantly higher in the dietary supplement groups than in the control group (**Figures 3A–D**; P < 0.05). However, the combination group (Q + VE) showed an elevated concentration of *occludin, ZO1, claudin 1*, and *Mucin 2* compared to the quercetin and vitamin E groups (**Figures 3A–D**; P < 0.05).

### Impacts of Quercetin, Vitamin E, and Q + VE on the Relative mRNA Expressions of Pro- and Anti-Inflammation Related Cytokines in the Small Intestinal Mucosa Tissues

**Figure 4:**
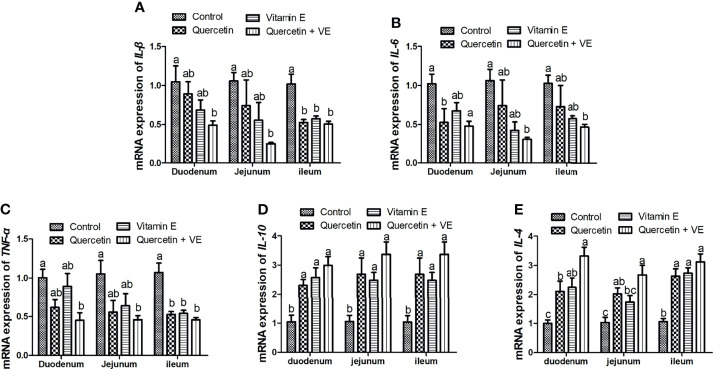
Effects of Q, VE, and Q + VE on the mRNA expressions of pro- and anti-inflammation related cytokines (*IL-6, IL-1β*, and *TFN-α; IL-10* and *IL-4*) related genes in the small intestinal mucosa of aged breeder hens. mRNA expressions of pro-inflammatory cytokines **(A–C)** and anti-inflammatory cytokines related genes **(D, E)**. Bars without the same letter differed significantly (P < 0.05).

**Figure 4** summarizes the relative mRNA expression of inflammation related cytokines in the intestinal mucosa. The levels of pro-inflammatory cytokines (*TNF-α, IL-6*, and *IL-1β*) were decreased in all the small intestinal segments due to the combinatory effects exerted by Q and VE as compared to all the other groups (**Figures 4A–C**; P < 0.05), whereas the combination of Q and VE significantly increased the levels of the anti-inflammatory cytokines (*IL-10* and *IL-4*) in the mucosa of the duodenum, jejunum, and ileum as compared to the control group (**Figures 4D, E**; P < 0.05). Moreover, in most cases, levels of the pro- and anti-inflammatory cytokines in the control, quercetin, and vitamin E groups were similar (**Figures 4A–E**; P > 0.05).

### Effects of Quercetin, Vitamin E, and Q + VE on the Relative mRNA Expressions of Antioxidant and Apoptosis Related Genes in the Small Intestinal Mucosa Tissues

**Figure 5:**
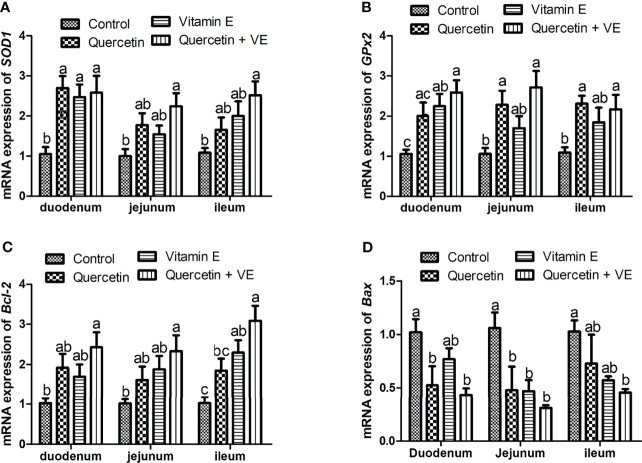
Effects of dietary Q, VE, and Q + VE on the mRNA expressions of antioxidant and apoptosis related genes in the small intestine mucosa of aged breeder hens. mRNA expressions of antioxidant related genes **(A, B)** and apoptosis related genes **(C, D)**. Bars without the same letter differed significantly (P < 0.05).

As shown in **Figure 5**, the dietary combination of Q + VE significantly increased the mRNA expression of the antioxidant related genes (*SOD1* and *GPx2*) in the mucosa of the small intestine of the aged chickens as compared to the other groups (**Figures 5A, B**; P < 0.05). Furthermore, Q, VE, and Q + VE significantly increased the concentration of the anti-apoptotic gene (*Bcl-2*) and decreased the expression of *Bax* in the intestinal mucosa of the aged chickens compared to the control (**Figures 5C, D**; P < 0.05).

### Effects of Quercetin, Vitamin E, and Q + VE on the Intestinal Damage of Aged Breeder Hens

**Figure 6:**
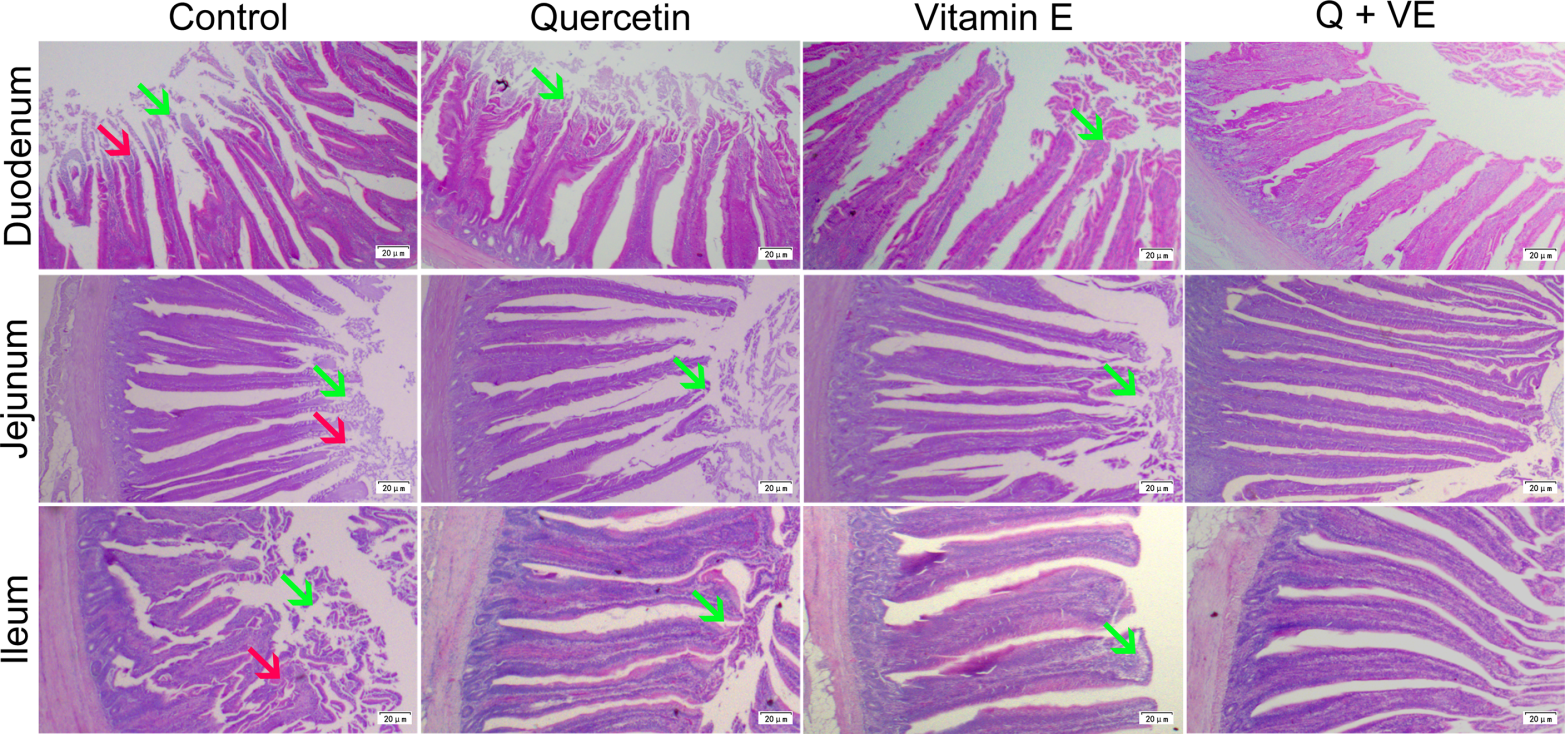
Effect of Q, VE, and Q + VE on intestinal inflammation in aged breeder hens. (Control, Q, and VE) Effects of dietary Q, VE, and Q + VE on intestinal histology in the Control group; quercetin group; vitamin E group; and Q + VE groups.

As shown in **Figure 6**, the histomorphological results showed that the duodenum, jejunum, and ileum of the aged hens in the control group exhibited shortening villi and infiltration of inflammatory cells, characterized with lymphocytes neutrophils, and macrophages (**Figure 6**, Control). However, in the individual quercetin and vitamin E groups, there were mild lesions observed in the duodenal, jejunal, and ileal tissue samples of the aged hens (**Figure 6**, quercetin and vitamin E). Moreover, the combination of quercetin and vitamin E (Q + VE) mitigated the intestinal damage induced by aging (**Figure 6**, Q + VE).

**Figure 7:**
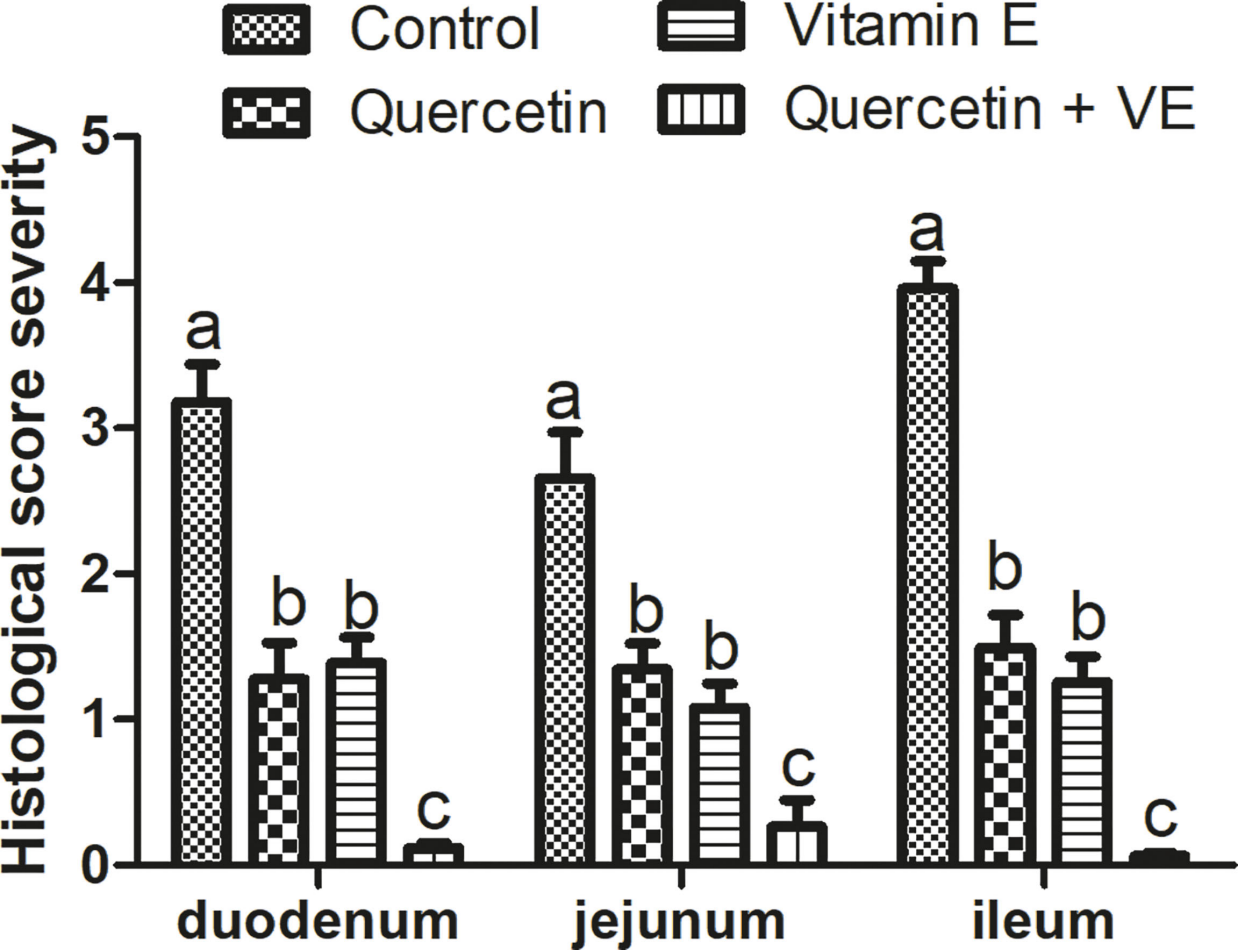
Histopathological severity scores of the four (4) groups. Bars without the same letter differed significantly (P < 0.05). Scale bar = 20um.

In addition, the histopathological scores obtained were consistent with the histological evaluation (**Figure 7**; P < 0.05). These results showed that the dietary combination of Q + VE could synergistically prevent intestinal structure damage by attenuating intestinal inflammation in aged breeder hens.

### Effects of Dietary Q, VE, and Q + VE on the Characteristics of the Intestinal Morphology of Aged Breeder Hens

The results presented in **Table 5** show that the villi height (VH) and crypt depth (CD) of the duodenal, jejunal and ileal sections of the small intestine in the Q, VE, and Q + VE groups were significantly higher than those in the control group (P < 0.05). However, there was no significant difference observed among the individual Q, VE, and control groups across all the small intestinal segments (P > 0.05). Therefore, this result suggested that, the synergism of Q + VE could improve the structure of the duodenum, jejunum, and ileum of aged breeder hens.

**Table 5:** Duodenal, jejunal, and ileal morphological characteristics of aged breeder chickens fed with Q, VE, and Q + VE.

Segments	Treatments					
	Parameters	Control	Q	VE	Q + VE	P-value
** Duodenum**	Villi height, µm	666.24^b^ ± 56.56	819.24^ab^ ± 38.79	750.76^ab^ ± 42.65	912.25^a^ ± 45.88	0.004
	Crypt depth, µm	152.55^b^ ± 6.46	159.19^ab^ ± 5.69	155.40^ab^ ± 10.02	192.80^a^ ± 12.83	0.010
** Jejunum**	Villi height, µm	647.16^b^ ± 39.72	700.52^ab^ ± 39.31	688.43^ab^ ± 37.22	789.86^a^ ± 33.82	0.064
	Crypt depth, µm	149.34^b^ ± 6.61	155.08^ab^ ± 6.71	155.09^ab^ ± 8.31	190.63^a^ ± 9.45	0.003
** ileum**	Villi height, µm	702.90^b^ ± 28.81	841.25^ab^ ± 34.19	822.11^ab^ ± 38.82	934.85^a^ ± 92.58	0.037
	Crypt depth, µm	145.56^b^ ± 6.13	159.72^ab^ ± 10.48	153.99^ab^ ± 8.35	202.00^a^ ± 11.56	0.001

^ab^Means within 4 treatments (Control, Q, VE, and Q + VE) lacking a common superscript differed significantly (P < 0.05).

## Discussion

The benefits of the intestinal health in animal wellbeing and welfare is unmeasurable. The importance of gut health in animal welfare and wellbeing is undisputable because of its important physiological functions (25). The current study elucidated the synergistic effects of dietary supplementation of quercetin and vitamin E on the intestinal structure and barrier integrity of aged breeder hens to promote immune response, gut homeostasis, and intestinal health. The results obtained in this study showed that the combination of quercetin and vitamin E promoted the palatability of the diet which promoted feed intake. In aged animals, the integrity of the intestinal barrier is compromised by pathogens (62–64), and these pathogens release certain biomarkers such as D-Lactate into the blood after gut damage. Plasma biomarkers such as DAO and D-Lac have been used as indicators of intestinal mucosal mass and integrity. DAO is an intracellular enzyme abundant in the epithelium of the small intestine (65), whereas, D-Lac is a product of intestinal bacteria (66) released into the blood during villi injury. Therefore, DAO and D-Lac are biomarkers used to assess gut permeability and mucosal damage (65).

In this study, we found that the combination of quercetin and vitamin E synergistically decreased plasma DAO and D-Lactate levels, indicating that intestinal mucosal integrity was protected by the attenuation of intestinal permeability. This was consistent with the study by Batista et al. (53), who reported that quercetin treatment significantly decreased plasma DAO activity, D-Lactate, and endotoxin levels in mice (53).

The intestinal mucosa is the first line of defense against oxidative stress, because it is composed of an extensive enzyme and non-enzymatic antioxidant system. However, in aged animals, due to imbalances in the antioxidant system, the intestinal mucosa experiences oxidative stress (25, 54). Previous studies have shown that aged animals fed without dietary antioxidant supplements experience oxidative stress responses (26, 54).

In this study, the antioxidant capacity was improved by the combination of quercetin and vitamin E in the intestinal mucosa and serum of aged breeder hens. Similar studies have shown that individual quercetin and vitamin E supplementation prevented the production and accumulation of ROS induced by proinflammatory cytokines; thereby, alleviating oxidative stress and apoptosis in animals (25, 43, 54, 67, 68).

In poultry, intestinal health and function are widely evaluated based on the characteristics of the gut morphological parameters such as villus height, crypt depth, and ratio of villus height to crypt depth (69–72). Longer villi length, deeper crypts, and a higher ratio of villus height to crypt depth increase nutrient absorption capacity, due to the large surface area (72–75). Thus, the higher the villi height, the higher the surface area for nutrient absorption in the small intestine. This process increases the action of digestive enzymes, and speeds up nutrient transportation (73, 76). Furthermore, the intestinal crypts are invaginations of the epithelium around the villi, and are lined by epithelial cells that secrete enzymes. The base of the crypts constantly divides to maintain the structure of the villi, therefore, an increase in crypt depth would produce more developed villi (73, 77, 78). Deeper crypts alternatively indicate fast tissue turnover because various types of special cells are present in the crypt, including absorptive, secretory and regenerative cells (79). Moreover, reducing the crypt depths of the intestinal villi may lead to a reduction in the absorption of nutrients (76, 78, 80). In this study, the villi height and crypt depth of the duodenum, jejunum, and ileum were enhanced by the combination of quercetin and vitamin E, indicating an improved absorption capacity in aged chickens. This was consistent with previous studies, which reported that quercetin significantly increased the depth of intestinal glands in rats and chickens, thereby improving intestinal morphology (52, 54, 81). Similarly, Wang et al. (26) reported that, vitamin E in combination with alpha lipoic acid promoted intestinal morphological characteristics in chickens (26). Therefore, the current study showed that the combination of dietary combination of quercetin and vitamin E promoted the epithelial structure of aged chickens.

The intestinal mucosa is made up of the membrane-bound and secreted mucins including Mucin 2 (*MUC2*) (82). *MUC2* is responsible for preventing the internal invasion of pathogens, toxins, and foreign materials. Quercetin was reported to promote the upregulation of *MUC2* through the protein kinase C alpha/extracellular regulated protein kinases 1-2 pathway (54). A study by Damiano et al. (83) showed that quercetin induces mRNA levels of *MUC2* and *MUC5AC via PKCα/ERK1-2* pathway in human intestinal epithelial Caco-2 cells. Hence, it shows that quercetin exerts protective effects on the intestinal mucosal barrier through regulating molecular mechanism that maintain secretory function of the intestinal goblet cells and mucin levels in the enterocytes of human (83). In addition, sIgA, a predominant immunoglobulin in the mucosal system, protects the mucosal surface against toxins, viruses, and enteropathogens; as well as inhibit pathogens from binding to the mucosal surface of the intestine, thereby enhancing immunity (84, 85). The results obtained in this study showed that the combination of quercetin and vitamin E promotes sIgA levels and mRNA expression of *MUC2* in the intestinal mucosa, thereby improving intestinal immunity.

Tight junction proteins including *claudin 1*, *ZO1*, and *occludin* promote normal functioning of the intestinal mucosa barrier. The expression of these proteins is downregulated by conditions such as “Leaky gut’’ or inflammation (86–89), therefore, tight junction proteins are crucial for determining paracellular permeability (90). The results of the present study showed that the combination of quercetin and vitamin E increased the mRNA expression of c*laudin 1*, *occludin*, and *ZO1* in the duodenum, jejunum, and ileum of aged chickens. This indicated that the combination dietary between quercetin and vitamin E exerted intestinal homeostasis and immunity in aged chickens. This was consistent with the results of Valenzano et al. (44), Suzuki and Hara (45), and Dong et al. (54).

Cytokines, are endogenous mediators of the immune system and are responsible for controlling the occurrence of inflammatory reactions (74, 91–93). The intestinal barrier regulates the passage of microorganisms, pro-inflammatory molecules, antigens, and toxins (94), however, age-related changes induce oxidative stress, which eventually increases pro-inflammatory cytokines (95), resulting in intestinal epithelial cell damage and cellular apoptosis (96–98).

Dietary antioxidants suppress pro-inflammatory enzymes, thereby attenuating intestinal inflammation (99). In this study, the combination of dietary quercetin and vitamin E reduced the expression of pro-inflammatory cytokines, increased the expression of anti-inflammatory cytokines, decreased apoptosis, and attenuated oxidative stress in the small intestinal structure, thereby promoting immunoregulation in aged breeder hens. The expression levels of the apoptotic gene (*Bax)* in this study was reduced significantly, whereas *Bcl-2* was significantly increased in the intestinal tissues of the chickens fed the combination of dietary quercetin and vitamin E. These results are consistent with previous studies that reported that a balanced enzymatic antioxidant system attenuates oxidative stress and hence, alleviates apoptosis; also dietary antioxidants such as quercetin reduces apoptosis in birds (100–102). This could be attributed to the beneficial effects of quercetin and vitamin E on the gut histomorphological results obtained. This was consistent with previous studies by Uyanga et al. (25), Abedi et al. (68), Yang et al. (102) and Shu et al. (42) who reported that dietary antioxidant supplementation improves immunity and antioxidant capacity in animals. Moreover, studies by Wang et al. (26) and Lewis et al. (103) showed that vitamin E supplementation positively mitigates intestinal inflammation and improves nutrient transport in chickens and mice, by suppressing pro-inflammatory cytokines and prostaglandin E2 (26, 103).

## Conclusions

In summary, the synergy between Quercetin and Vitamin E attenuated age-induced intestinal permeability, inflammation, and oxidative stress by promoting the activities of intestinal antioxidant biomarkers, anti-inflammatory cytokines, and elevation of intestinal tight junction proteins; as well as mRNA expression of genes related to improving the intestinal integrity and structure in aged breeder hens. Taken together, these findings provide novel insights into the use of a combination of dietary Quercetin and Vitamin E as anti-aging agents in the development of therapeutic and preventive strategies for age-induced intestinal barrier disruption in aged breeder hens.

## Data Availability Statement

The original contributions presented in the study are included in the article/supplementary material. Further inquiries can be directed to the corresponding author.

## Ethics Statement

The animal study was reviewed and approved by Animal Care and Use Committee of Sichuan Agricultural University, China. Animals used in this experiment were cared for under the guidelines stated in the Guide for the Care and Use of Agricultural Animals in Agricultural Research and Teaching of Sichuan Province, China (No. 2019502005).

## Author Contributions

The authors’ contributions are as follows: FKA, XZ, and GS designed and conceived this study. FKA, ZC, and XD conducted the experiments. FKA, ZC, XD, ZN, XD, DX, GS, YHW, XC, WS, and YT collected the samples and performed the analysis of samples. FKA, ZC, DL, YW, YZ, XHD, QZ, and XH analyzed the data. FKA wrote the manuscript. FA, XZ, QZ, DL, YW, and XHD revised and edited the manuscript. All authors contributed to the article and approved the submitted version.

## Funding

The authors thank National Natural Science Foundation of China (Grant No. 31872347), the Science and Technology Innovation and Entrepreneurship Seedling Project of the Sichuan Science and Technology Program (2020JDRC0104), the Key Research & Development Plan of the Department of Science and Technology of Tibet Autonomous Region (XZ202101ZY0002N), the Local Projects Guided by the Central Government from Razi County, Tibet Autonomous Region, and the Projects Funded by the Central Government to Guide Local Scientific and Technological Development from Guizhou province (QIANKEZHONGYINDI[2021]4003) for funding this work.

## Conflict of Interest

The authors declare that the research was conducted in the absence of any commercial or financial relationships that could be construed as a potential conflict of interest.

## Publisher’s Note

All claims expressed in this article are solely those of the authors and do not necessarily represent those of their affiliated organizations, or those of the publisher, the editors and the reviewers. Any product that may be evaluated in this article, or claim that may be made by its manufacturer, is not guaranteed or endorsed by the publisher.
